# A statistical evaluation of the effects of gender differences in assessment of acute inhalation toxicity

**DOI:** 10.1177/0960327110370982

**Published:** 2010-03

**Authors:** Charlotte Price, Nigel Stallard, Stuart Creton, Ian Indans, Robert Guest, David Griffiths, Philippa Edwards

**Affiliations:** 1 Warwick Medical School, University of Warwick, Coventry, UK; >2 National Centre for the Replacement, Refinement and Reduction of Animals in Research (NC3Rs), London, UK; 3 Health Directorate, Health and Safety Executive, Bootle, Merseyside, UK; 4 Harlan Laboratories Ltd, Shardlow, UK; 5 Health Protection Agency, Didcot, UK

**Keywords:** acute inhalation toxicity, OECD test guidelines, fixed concentration procedure, gender differences

## Abstract

Acute inhalation toxicity of chemicals has conventionally been assessed by the median lethal concentration (LC_50_) test (organisation for economic co-operation and development (OECD) TG 403). Two new methods, the recently adopted acute toxic class method (ATC; OECD TG 436) and a proposed fixed concentration procedure (FCP), have recently been considered, but statistical evaluations of these methods did not investigate the influence of differential sensitivity between male and female rats on the outcomes. This paper presents an analysis of data from the assessment of acute inhalation toxicity for 56 substances. Statistically significant differences between the LC_50_ for males and females were found for 16 substances, with greater than 10-fold differences in the LC_50_ for two substances. The paper also reports a statistical evaluation of the three test methods in the presence of unanticipated gender differences. With TG 403, a gender difference leads to a slightly greater chance of under-classification. This is also the case for the ATC method, but more pronounced than for TG 403, with misclassification of nearly all substances from Globally Harmonised System (GHS) class 3 into class 4. As the FCP uses females only, if females are more sensitive, the classification is unchanged. If males are more sensitive, the procedure may lead to under-classification. Additional research on modification of the FCP is thus proposed.

## Introduction

Acute systemic toxicity studies based on the determination of a median lethal dose (LD_50_), that is the single dose of a substance that can be expected to kill 50% of the animals in a test group, were first proposed by Trevan in 1927 for the purposes of ranking the toxicity of substances intended for human use.^1^ Since this time, LD_50_ tests have gained general acceptance as a means of comparing and classifying the toxicity of chemicals and have become a routine test requirement under a number of regulatory frameworks. Originally, the test required up to 100 animals for each substance tested, but over the last few decades, alternative methods have been developed that have significantly reduced and refined animal use, particularly for testing by the oral route.^2^

For acute inhalation toxicity, the internationally accepted test method has been the median lethal concentration (LC_50_) test in rodents, usually rats, outlined in organisation for economic co-operation and development (OECD) test guideline (TG) 403.^3^ The procedure uses death, or impending death, as the indicator of toxicity and follows a similar strategy to the now deleted OECD TG 401 for acute oral toxicity.^4^ It was designed to identify the LC_50_ of a substance, that is the concentration that can be expected to cause death in 50% of the animal population, where ‘death’ is used throughout this paper to mean compound-related mortality within 14 days. A major use of the estimated LC_50_ arising from such tests is the assignment of the test substance into a particular toxic class for the purpose of classification and labelling. Table 1 shows the classifications for vapours, dusts and mists and gases under the Globally Harmonised System of Classification and Labelling of Chemicals (GHS),^5^ which was devised at a time when TG 403 was the only internationally recognized test method for this endpoint. Although alternative ‘up and down’ methods for the estimation of oral LD_50_ exist,^6–8^ the challenge of exposure at more than a small number of distinct concentrations makes these less suitable for the assessment of toxicity via the inhalation route.

**Table 1.** GHS classifications for LC50 by inhalation

GHS class	Vapours (mg/L)	Dusts and mists (mg/L)	Gases (ppm)
1	≤0.5	≤0.05	≤100
2	>0.5 and ≤2	>0.05 and ≤0.5	>100 and ≤500
3	>2 and ≤10	>0.5 and ≤1	>500 and ≤2500
4	>10 and ≤20	>1 and ≤5	>2500 and ≤20000
5	>20	>5	>20000

GHS, Globally Harmonised System; LC_50_, median lethal concentration; ppm, parts per million.

OECD TGs are periodically reviewed in light of scientific progress and animal welfare considerations and two alternative testing procedures for inhalation toxicity, a revised TG 403^9^ and the acute toxic class (ATC) method for inhalation exposure (OECD TG 436^10^), have recently been published on the OECD website (www.oecd.org). The revised TG 403 includes two study types, the traditional LC_50_ protocol and a concentration × time (C × T) protocol. The latter is for use when there is a specific regulatory or scientific need to assess the relationship between exposure time and concentration on toxicity. The ATC method has advantages over TG 403 in that fewer animals are used (a maximum of 24 compared to a maximum of 40 for the LC_50_ protocol) and the pre-specification of experimental pathways (sequential choice of pre-set concentrations) facilitates the execution of the protocol in the laboratory.^11^

A further alternative procedure for acute inhalation testing, the fixed concentration procedure (FCP; draft OECD TG 433),^12^ which is similar to the fixed dose procedure for acute oral toxicity (TG 420),^13^ is currently under development. Compared to the TG 403 methods, the FCP exposes far fewer animals (rarely more than 10).^14^ It also provides a refinement over TG 403 and the ATC method as it uses non-lethal toxicity as an endpoint rather than death, thereby reducing suffering. A statistical evaluation of the FCP by Stallard et al.^14^ found that, for classifications made according to the GHS, substances are likely to be assigned either to the class corresponding to the LC_50_ value or to a more toxic class. Concern that this would lead to over-classification was one of the reasons why the progression of the FCP through the OECD adoption process was suspended whilst further work was carried out. A further concern was that the FCP tests only one gender, whereas the LC_50_ method and the ATC method test both genders, unless there is prior evidence to show that one gender is more susceptible than the other.

The suitability of the LC_50_, or related estimates of concentrations that are lethal to animals, for assessing the risks of adverse effects in humans has been questioned.^15–17^ However, for the present, it is the internationally accepted basis for classification and labelling of substances for acute toxicity. In order to achieve international acceptance, it is necessary that any new procedure for estimating acute inhalation toxicity provides data that can be used for this purpose. The UK National Centre for the Replacement, Refinement and Reduction of Animals in Research (NC3Rs) is coordinating a collaborative project to develop the scientific evidence base needed to support the adoption of the FCP as an approved test method. This paper reports part of this work, providing a detailed statistical analysis of the performance of the FCP in comparison to the performances of the other available methods. To date, evaluations of test methods for acute inhalation toxicity have not taken into account the possible influence on test performance of differences in the susceptibility of males and females to acute inhalation exposure. It has been reported that there are, in general, limited gender differences in acute oral toxicity studies and that where differences exist, females are often more sensitive.^18,19^ However, there is little information available on the relative sensitivity of males and females in acute inhalation testing.^20^ To address this, historical data were analyzed to assess the potential for gender differences that can arise in acute inhalation toxicity. Gender differences of the magnitude indicated were then included in the statistical comparison of the test methods. This study provides data that can be used to evaluate whether the FCP can be considered as reliable as the other two approaches for the purpose of classification, and the extent to which testing in a single gender affects reliability.

## Methods

### LC_50_ method (TG 403)

Test guidelines for the LC_50_ method state that at least 10 animals (five males and five females) should be exposed at each of at least three concentration levels.^3^ The concentration levels should be sufficiently spaced to enable a concentration mortality curve to be produced and an estimate of the LC_50_ to be obtained. In practice, the LC_50_ value is mainly used for classification into one of the GHS classes indicated in Table 1. The GHS classes are defined by ranges of LC_50_ values that vary in size. For example, for dusts and mists, there is a ten-fold range of LC_50_ values in class 2, a two-fold range in class 3 and a five-fold range in class 4.

**Figure 1.**
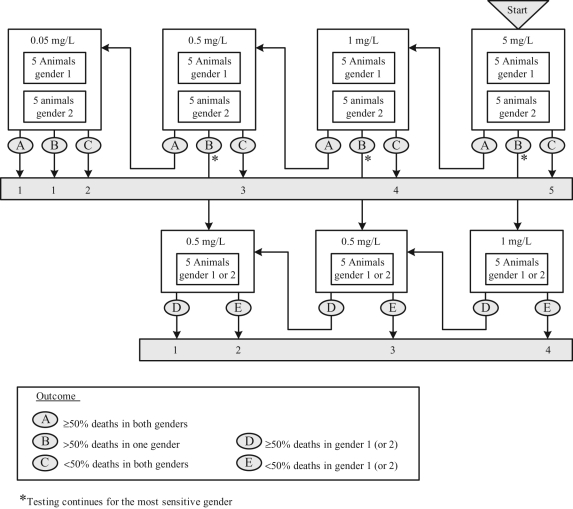
LC_50_ test (OECD test guideline 403) for dusts and mists starting at 5 mg/L.

When used for classification, the test often begins with a group of 10 animals exposed at a concentration corresponding to the lower limit of the least toxic class and proceeds in a stepwise manner to subsequently expose groups of 10 animals at lower concentrations until a classification can be made. This is achieved when mortality is seen in less than 50% of the males and less than 50% of the females or when the concentration corresponding to the LC_50_ boundary for the most toxic class of chemicals is reached. This procedure is illustrated in Figure 1 .

A similar procedure can also be envisaged by selecting a starting concentration to correspond to the upper limit of one of the GHS classes considered likely to lead to death in some of the animals. If death is observed in more than 50% of either the males or the females, testing continues at successively lower concentrations until less than 50% of males and less than 50% of females die, or testing occurs at the lowest concentration, in which case the substance is classified into the most toxic class. If death is observed in less than 50% of both males and females, testing continues at successively higher concentrations until more than 50% of either males or females die, or testing occurs at the highest concentration.

### Acute toxic class method (TG 436)

**Figure 2.**
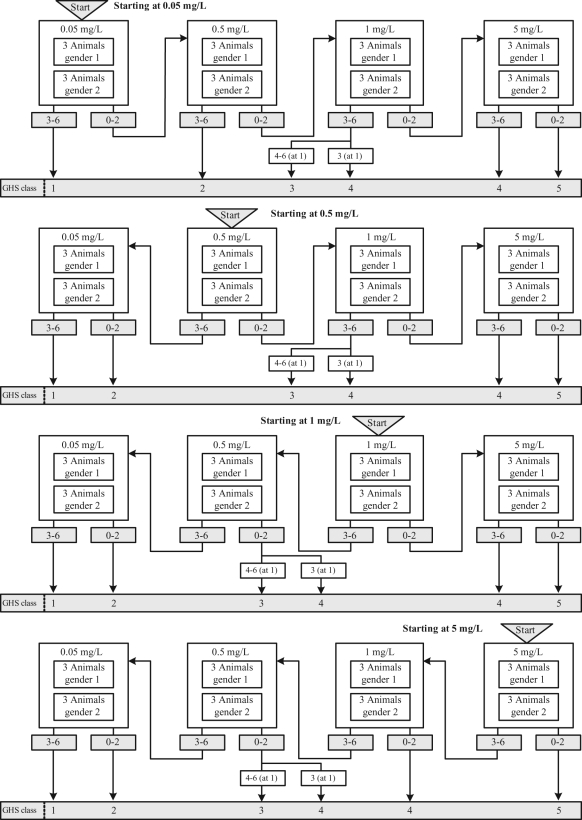
Acute toxic class (ATC) method for dusts and mists.

The ATC method,^10^ as illustrated in Figure 2 , is a stepwise procedure that tests three males and three females at each step. A starting concentration is chosen from one of the four fixed concentrations that form the upper limits of the GHS classes, 0.05, 0.5, 1 and 5 mg/L for dusts and mists, and should be either the highest concentration or that which is expected to lead to mortality in some of the exposed animals, based on prior information. The guideline recommends testing in six animals of the most sensitive gender only when there is evidence to suggest that one gender is more susceptible than the other, although no indication is given as to what would comprise such evidence. At each step, decisions are based on the number of observed deaths from the combined group of six animals and either a classification is made or testing continues at the next higher or lower concentration, depending on the starting concentration. Mortality guides the process and determines when testing stops and the substance can be classified. A statistical evaluation of the ATC method for acute oral toxicity can be found in Stallard and Whitehead.^21^

### Fixed Concentration Procedure (draft TG 433)

**Figure 3.**
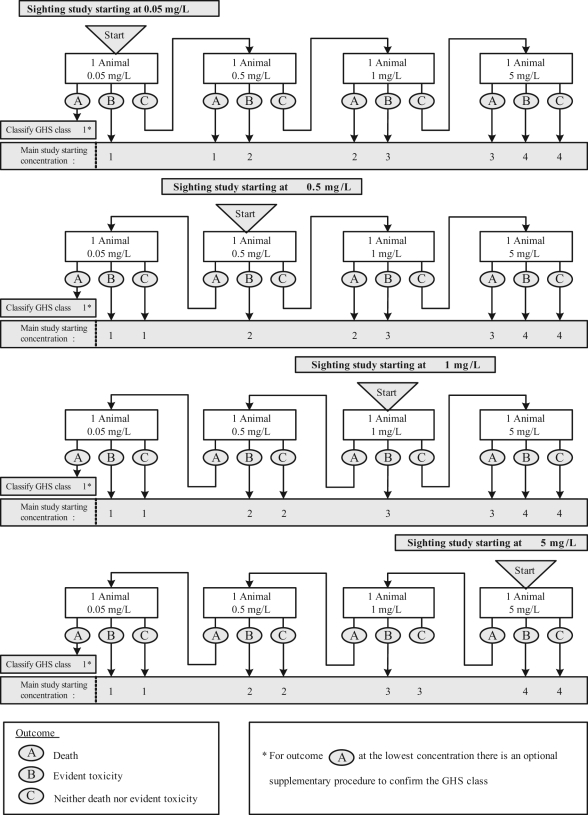
Fixed concentration procedure (FCP) sighting study for dusts and mists.

**Figure 4.**
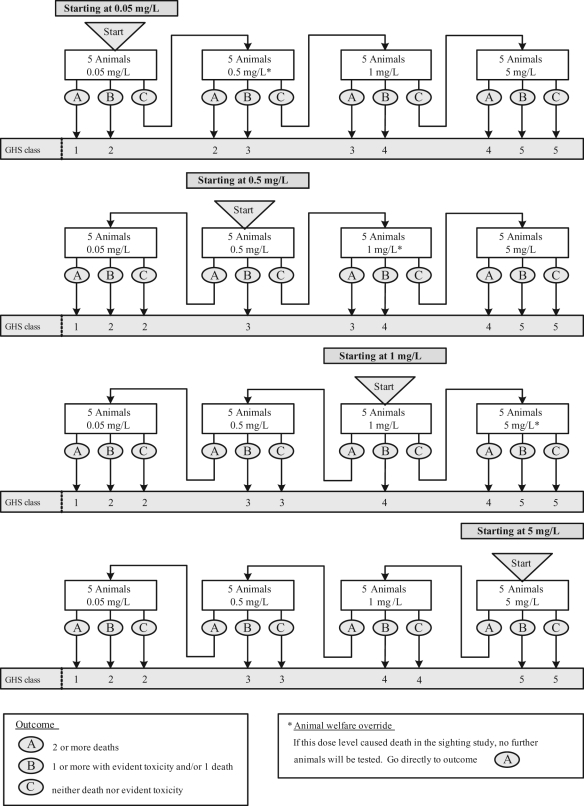
Fixed concentration procedure (FCP) main study for dusts and mists.

Unlike the LC_50_ and ATC methods, in the FCP, animals of a single gender should be exposed to the test substance at one or more of the four fixed concentrations that form the upper limits of the GHS classes. The procedure uses females, unless there is prior evidence to suggest that males are more susceptible,^12^ and starts with a sighting study in which single animals are exposed sequentially to one or more of the fixed concentrations (Figure 3 ). The starting concentration for the sighting study is chosen to be the fixed concentration level that is most likely to lead to evident toxicity but not death, that is clear signs of toxicity such that it can be predicted that exposure to the next highest concentration would cause severe toxicity or death in most animals.^14^ If death occurs at the lowest concentration level, the substance is classified into the most toxic class and a main study is not needed. Otherwise, the sighting study is followed by a main study in which groups of five animals are exposed at each concentration level until a classification can be made (Figure 4 ).

### Limit tests

If information is available indicating that the test substance is likely to be non-toxic, a limit test may be used whereby the study is performed in a single group of animals using one limit concentration, generally selected on the basis of regulatory requirements. Under the GHS scheme, limit concentrations for gases, vapours and dusts/mists are 20,000 parts per million (ppm), 20 mg/L and 5 mg/L, respectively. In the sequential TG 403 method described, if testing starts at the highest concentration and leads to no compound-related mortality, a full study may not be needed, with this single exposure counting as a limit test. A similar outcome is obtained with the ATC method and the FCP if testing starts at the highest concentration and no compound-related mortality is observed, since classification then follows from the observed results at this single concentration. As such, if testing starts at the highest concentration and no compound-related mortality is observed, TG 403, the ATC method and the FCP all result in a limit test for the least toxic substances, with the use of ten, six and six animals (one in the sighting study and five in the main study), respectively.

### Assessment of gender differences in sensitivity to acute inhalation exposure

A statistical analysis was carried out to address the potential for gender differences in the sensitivity of rats to acute inhalation toxicity using data from tests conducted according to TG 403, which are available in Annex 5 of the 2008 Performance Assessment Report.^11^ The database provides details of 168 studies, including the concentration levels at which testing occurred (mg/L), number of rats tested at each level, incidence of death and, in some but not all cases, an estimated LC_50_ for the test substance based on the observed data.

The analyses were carried out on individual studies rather than individual substances, which means that different studies of the same substance were analysed separately. A study was excluded from the analysis if it had incomplete gender and/or substance concentration information, or if it was conducted as a limit test which showed no lethality at the top (limit) concentration. After exclusions, the data from 84 studies were analysed to compare the LC_50_ of the two genders.

Statistical analysis was carried out using probit regression, including terms for gender and the log (to base 10) of the concentration but no interaction between exposure concentration and gender. The inclusion of an interaction term in the statistical model was investigated for each study in the database but was found to be not significant in all cases.

### Statistical evaluation of test methods

Stallard et al.^14^ proposed a statistical method for evaluating the performance of the FCP without differences in the sensitivity of males and females to acute inhalation exposure. A similar approach is adopted here to assess the classification performances of the LC_50_ method, the ATC method and the FCP, both with and without gender differences, thus allowing for a like-for-like comparison of the three test procedures.

For each of the three test procedures, the statistical method enables the calculation of the probability of classification into each toxic class for a range of hypothetical substances with specified properties, namely the LC_50_, concentration-response curve slope and, for the FCP, the TC_50_, where this is the concentration expected to cause death or evident toxicity in 50% of the animals. The method assumes that both the probability of death and the probability of either death or non-fatal evident toxicity are given by a concentration-response curve of the probit form. Based on these concentration-response curves, calculations can be performed to obtain the probability of each possible outcome at each test concentration. From this, the probability of classification into each toxic class can be calculated for the substance considered, along with the average number of animals required by the procedure and the number of deaths. If a gender difference is assumed, the model includes separate concentration-response curves for males and females with different LC_50_ values but the same slope. Further details are given in the Appendix.

In order to evaluate TG 403, it was necessary to make some assumptions about how the test would be conducted. It was assumed that testing is performed sequentially, as illustrated in Figure 1, or using a similar sequential procedure starting at a concentration selected to correspond to the upper limit of one of the more toxic GHS classes. Since TG 403 makes use of both male and female animals, and classifications are based on the classification for the more sensitive gender, no modifications were needed to evaluate the procedure in the presence of a gender difference to acute inhalation toxicity.

The FCP TG states that females should be used unless there is prior evidence that males are likely to be more susceptible. If females are indeed more sensitive than males, the performance of the FCP is unaffected by the gender difference since classification is based on the more sensitive gender. However, if males are more sensitive than females, and this is not anticipated, classification is then based on the less sensitive gender. The effect of this is evaluated below.

Unlike the FCP, the ATC method tests both males and females, and classifications are based on the total number of deaths from the combined group of animals. The guideline suggests that testing should be conducted in the more sensitive gender alone if a gender difference is indicated. In the evaluation reported below, it is assumed that no gender difference is suspected during the test procedure, so that testing continues in both genders throughout.

**Figure 5.**
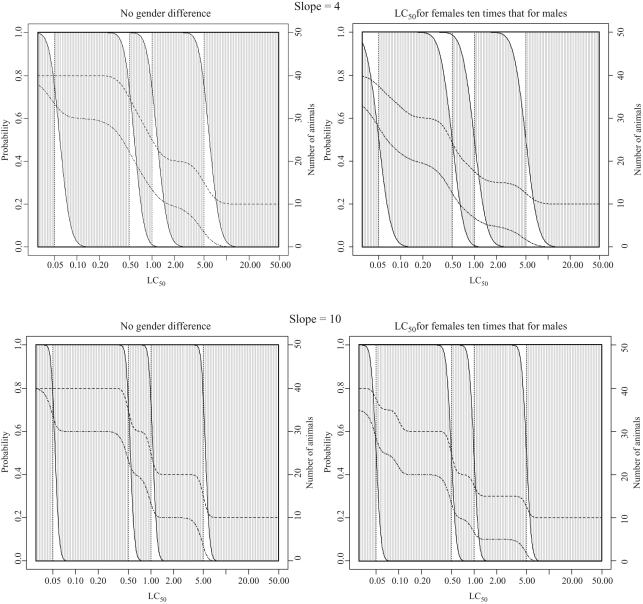
Classification probabilities and expected numbers of animals for test guideline (TG) 403 starting at 5 mg/L with concentration-response curve slopes of 4 and 10. Cumulative probabilities of classification (on left-hand axis scale) into each toxic class for LC_50_ values are shown. The height of the shaded areas gives the probability of correct classification, the height of the area below the shaded area is the probability of classification into too toxic a class and the height of the area above the shaded area is the probability of classification into a class that is not toxic enough. The dashed lines give expected number of animals and deaths (using the scale on the right-hand axis), with the top line indicating the number of animals used (see Results section for additional details).

**Figure 6.**
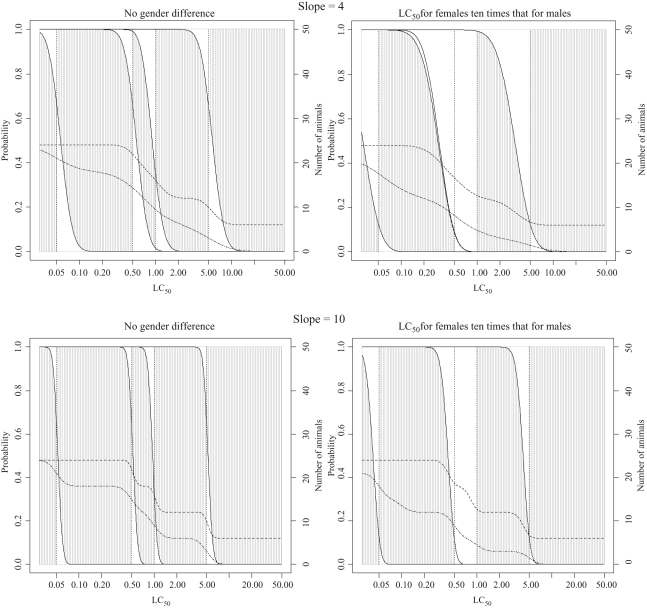
Classification probabilities and expected numbers of animals for the acute toxic class (ATC) starting at 5 mg/L with concentration-response curve slopes of 4 and 10 (see legend to Figure 5 and text of Results section for a detailed explanation of plotted lines and shaded regions).

**Figure 7.**
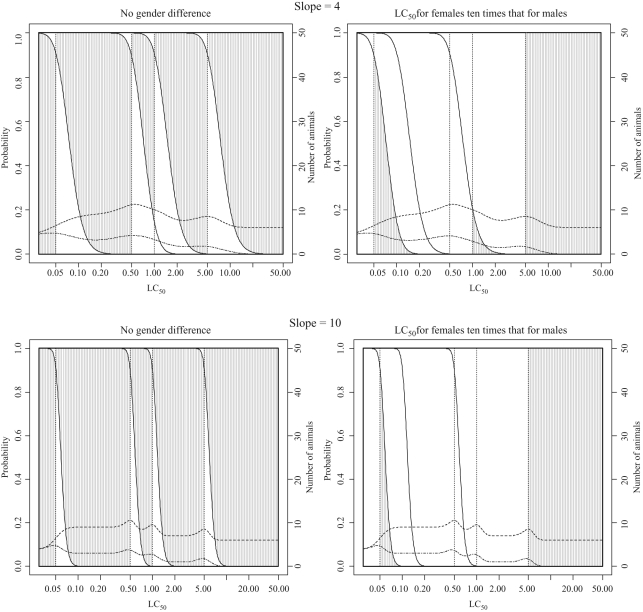
Classification probabilities and expected numbers of animals for the fixed concentration procedure (FCP) starting at 5 mg/L with concentration-response curve slopes of 4 and 10 (see legend to Figure 5 and text of Results section for a detailed explanation of plotted lines and shaded regions).

**Figure 8.**
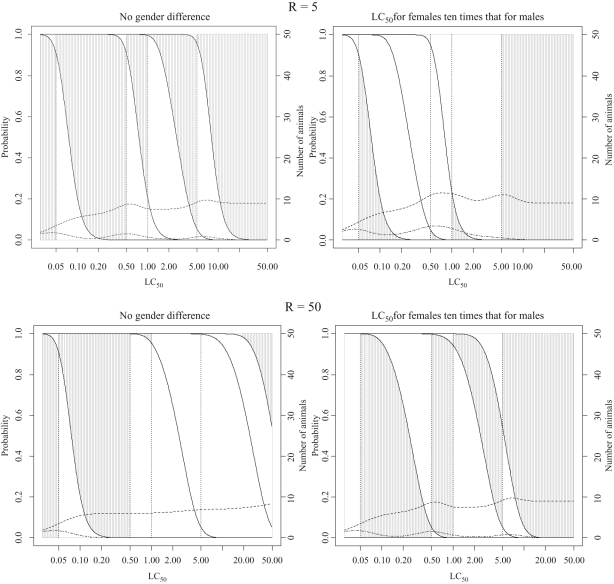
Classification probabilities and expected numbers of animals for the fixed concentration procedure (FCP) starting at 0.05 mg/L for substances with concentration-response curve slope = 4 and different values of *R* = LC_50_/TC_50_ (see legend to Figure 5 and text of Results section for a detailed explanation of plotted lines and shaded regions).

The procedures were evaluated for a range of hypothetical substances in the dusts and mists category. Two sets of results were obtained. The first set (shown in Figures 5–8) are for substances with LC_50_ values ranging from 0.01 to 50 mg/L, with starting concentrations of 5 mg/L and 0.05 mg/L for all procedures. The second set (shown in Tables 3–6) are for substances with LC_50_ values of 0.03, 0.15, 0.7, 1, 1.1, 2.5 and 10 mg/L, with starting concentrations of 0.05, 0.05, 0.05, 0.5, 0.5, 1 and 5 mg/L, respectively. These latter starting concentrations might be chosen if there was good prior knowledge of the LC_50_. In both cases, concentration-response curve slope values of 4 and 10 were considered. The latter is the mean (on the log scale) of the distribution of slopes used in the evaluation reported in ref 11, whilst under this distribution approximately 1% of substances would have a concentration-response curve slope less than 4. For the FCP, when using any starting concentration other than 5 mg/L, the classification depends on observation of evident toxicity as well as death. In this case, *R* values (i.e. the ratio of the LC_50_ to the TC_50_) of 5 and 50 were considered, and substances were also considered for which the concentration response curves for toxicity and lethality differed, with the slope for the toxicity curve equal to 4 and that for the lethality curve equal to 10.

**Table 3.** Properties of the LC50 method (OECD test guideline 403) for dusts and mists

LC_50_ identical for males and females (no gender difference)										
Substance				Classification probabilities					Estimated mean no. of animals	
LC_50_		Start concentration	Slope	Class 1	Class 2	Class 3	Class 4	Class 5	Tested	Deaths
0.03		0.05	4	**99.8**	0.2	0.0	0.0	0.0	10.0	8.0
0.15		0.05	4	0.0	**100.0**	0.0	0.0	0.0	20.0	9.8
0.70		0.05	4	0.0	25.5	**65.3**	1.1	0.0	27.6	6.1
1.00		0.50	4	0.0	2.5	**73.1**	24.4	0.0	22.3	5.9
1.10		0.50	4	0.0	1.1	60.7	**38.3**	0.0	23.8	6.3
2.50		1.00	4	0.0	0.0	0.3	**99.7**	0.0	20.0	8.8
10.00		5.00	4	0.0	0.0	0.0	2.5	**97.5**	10.1	0.1
0.03		0.05	10	**100.0**	0.0	0.0	0.0	0.0	10.0	9.9
0.15		0.05	10	0.0	**100.0**	0.0	0.0	0.0	20.0	10.0
0.70		0.05	10	0.0	0.7	**99.1**	0.0	0.0	29.9	9.3
1.00		0.50	10	0.0	0.0	**75.0**	25.0	0.0	22.5	5.9
1.10		0.50	10	0.0	0.0	39.0	**61.0**	0.0	26.1	7.5
2.50		1.00	10	0.0	0.0	0.0	**100.0**	0.0	20.0	10.0
10.00		5.00	10	0.0	0.0	0.0	0.0	**100.0**	10.0	0.0
LC_50_ for females 10 times greater than LC_50_ for males										
LC_50_ (M)	LC_50_ (F)									
0.03	0.30	0.05	4	**95.1**	4.9	0.0	0.0	0.0	10.5	4.4
0.15	1.50	0.05	4	0.0	**100.0**	0.0	0.0	0.0	20.0	4.9
0.70	7.00	0.05	4	0.0	13.7	**75.7**	10.6	0.0	29.7	4.0
1.00	10.00	0.50	4	0.0	1.2	**49.4**	49.4	0.0	24.9	4.2
1.10	11.00	0.50	4	0.0	0.5	37.6	**61.8**	0.0	26.2	4.4
2.50	25.00	1.00	4	0.0	0.0	0.2	**98.6**	1.2	20.0	4.4
10.00	100.00	5.00	4	0.0	0.0	0.0	1.2	**98.8**	10.1	0.0
0.03	0.30	0.05	10	**100.0**	0.0	0.0	0.0	0.0	10.0	4.9
0.15	1.50	0.05	10	0.0	**100.0**	0.0	0.0	0.0	20.0	5.0
0.70	7.00	0.05	10	0.0	0.3	**99.5**	0.2	0.0	30.0	4.7
1.00	10.00	0.50	10	0.0	0.0	**50.0**	50.0	0.0	25.0	4.2
1.10	11.00	0.50	10	0.0	0.0	21.9	**78.1**	0.0	27.8	4.6
2.50	25.00	1.00	10	0.0	0.0	0.0	**100.0**	0.0	20.0	5.0
10.00	100.00	5.00	10	0.0	0.0	0.0	0.0	**100.0**	10.0	0.0

LC_50_, median lethal concentration.

**Table 4.** Properties of the acute toxic class method (OECD test guideline 436) for dusts and mists

LC_50_ identical for males and females (no gender difference)										
Substance				Classification probabilities					Estimated mean no. of animals	
LC_50_		Start concentration	Slope	Class 1	Class 2	Class 3	Class 4	Class 5	Tested	Deaths
0.03		0.05	4	**98.7**	1.3	0.0	0.0	0.0	6.1	5.0
0.15		0.05	4	0.0	**100.0**	0.0	0.0	0.0	12.0	6.1
0.70		0.05	4	0.0	21.9	**62.6**	15.5	0.0	16.9	5.3
1.00		0.50	4	0.0	2.3	**33.6**	64.1	0.0	14.0	5.6
1.10		0.50	4	0.0	1.0	22.7	**76.2**	0.0	14.8	5.9
2.50		1.00	4	0.0	0.0	0.0	**99.8**	0.2	12.0	5.6
10.00		5.00	4	0.0	0.0	0.0	2.3	**97.7**	6.1	0.7
0.03		0.05	10	**100.0**	0.0	0.0	0.0	0.0	6.0	5.9
0.15		0.05	10	0.0	**100.0**	0.0	0.0	0.0	12.0	6.0
0.70		0.05	10	0.0	0.6	**99.0**	0.4	0.0	18.0	6.0
1.00		0.50	10	0.0	0.0	**34.4**	65.6	0.0	14.1	5.1
1.10		0.50	10	0.0	0.0	10.6	**89.4**	0.0	16.0	6.0
2.50		1.00	10	0.0	0.0	0.0	**100.0**	0.0	12.0	6.0
10.00		5.00	10	0.0	0.0	0.0	0.0	**100.0**	6.0	0.0
LC_50_ for females 10 times greater than LC_50_ for males										
LC_50_ (M)	LC_50_ (F)									
0.03	0.30	0.05	4	**53.8**	46.2	0.0	0.0	0.0	8.8	5.0
0.15	1.50	0.05	4	0.0	**95.1**	2.8	2.2	0.0	12.3	3.3
0.70	7.00	0.05	4	0.0	2.2	**0.0**	97.8	0.0	21.4	5.3
1.00	10.00	0.50	4	0.0	0.1	**0.0**	99.4	0.5	17.2	4.8
1.10	11.00	0.50	4	0.0	0.1	0.0	**99.0**	0.9	17.5	4.5
2.50	25.00	1.00	4	0.0	0.0	0.0	**69.7**	30.3	12.0	2.8
10.00	100.00	5.00	4	0.0	0.0	0.0	0.1	**99.9**	6.0	0.3
0.03	0.30	0.05	10	**96.1**	3.9	0.0	0.0	0.0	6.2	3.2
0.15	1.50	0.05	10	0.0	**100.0**	0.0	0.0	0.0	12.0	3.0
0.70	7.00	0.05	10	0.0	0.0	**0.0**	100.0	0.0	19.0	3.6
1.00	10.00	0.50	10	0.0	0.0	**0.0**	100.0	0.0	17.2	4.1
1.10	11.00	0.50	10	0.0	0.0	0.0	**100.0**	0.0	17.8	3.9
2.50	25.00	1.00	10	0.0	0.0	0.0	**99.6**	0.4	12.0	3.0
10.00	100.00	5.00	10	0.0	0.0	0.0	0.0	**100.0**	6.0	0.0

LC_50_, median lethal concentration.

**Table 5.** Properties of the fixed concentration procedure for dusts and mists (R = LC50/TC50 = 5)

LC_50_ identical for males and females (no gender difference)										
Substance				Classification probabilities					Estimated mean no. of animals	
LC_50_		Start concentration	Slope	Class 1	Class 2	Class 3	Class 4	Class 5	Tested	Deaths
0.03		0.05	4	**99.9**	0.1	0.0	0.0	0.0	1.9	1.6
0.15		0.05	4	3.5	**96.5**	0.0	0.0	0.0	6.1	0.4
0.70		0.05	4	0.0	58.6	**41.4**	0.0	0.0	8.5	1.3
1.00		0.50	4	0.0	20.5	**79.0**	0.5	0.0	6.7	0.7
1.10		0.50	4	0.0	14.1	84.7	**1.2**	0.0	6.6	0.6
2.50		1.00	4	0.0	0.0	8.2	**91.8**	0.0	6.4	0.5
10.00		5.00	4	0.0	0.0	0.0	20.6	**79.4**	6.6	0.6
0.03		0.05	10	**100.0**	0.0	0.0	0.0	0.0	1.1	1.1
0.15		0.05	10	0.0	**100.0**	0.0	0.0	0.0	6.0	0.0
0.70		0.05	10	0.0	11.3	**88.7**	0.0	0.0	7.2	0.4
1.00		0.50	10	0.0	0.1	**99.9**	0.0	0.0	6.0	0.0
1.10		0.50	10	0.0	0.0	100.0	**0.0**	0.0	6.0	0.0
2.50		1.00	10	0.0	0.0	0.0	**100.0**	0.0	6.0	0.0
10.00		5.00	10	0.0	0.0	0.0	0.1	**99.9**	6.0	0.0
LC_50_ for females 10 times greater than LC_50_ for males										
LC_50_ (M)	LC_50_ (F)									
0.03	0.30	0.05	4	**0.1**	99.8	0.1	0.0	0.0	7.2	1.0
0.15	1.50	0.05	4	0.0	**3.5**	87.2	9.3	0.0	7.5	0.4
0.70	7.00	0.05	4	0.0	0.0	**0.7**	67.5	31.9	9.7	0.8
1.00	10.00	0.50	4	0.0	0.0	**0.0**	24.3	75.7	8.3	0.5
1.10	11.00	0.50	4	0.0	0.0	0.0	**16.5**	83.5	8.2	0.4
2.50	25.00	1.00	4	0.0	0.0	0.0	**0.3**	99.7	7.0	0.0
10.00	100.00	5.00	4	0.0	0.0	0.0	0.0	**100.0**	6.0	0.0
0.03	0.30	0.05	10	**0.0**	100.0	0.0	0.0	0.0	6.8	0.8
0.15	1.50	0.05	10	0.0	**0.0**	98.7	1.3	0.0	7.0	0.0
0.70	7.00	0.05	10	0.0	0.0	**0.0**	13.0	87.0	9.1	0.3
1.00	10.00	0.50	10	0.0	0.0	**0.0**	0.1	99.9	8.0	0.0
1.10	11.00	0.50	10	0.0	0.0	0.0	**0.0**	100.0	8.0	0.0
2.50	25.00	1.00	10	0.0	0.0	0.0	**0.0**	100.0	7.0	0.0
10.00	100.00	5.00	10	0.0	0.0	0.0	0.0	**100.0**	6.0	0.0

LC_50_, median lethal concentration.

**Table 6.** Properties of the fixed concentration procedure for dusts and mists (R = LC50/TC50 = 50)

LC_50_ identical for males and females (no gender difference)										
Substance				Classification probabilities					Estimated mean no. of animals	
LC_50_		Start concentration	Slope	Class 1	Class 2	Class 3	Class 4	Class 5	Tested	Deaths
0.03		0.05	4	**99.9**	0.1	0.0	0.0	0.0	1.9	1.6
0.15		0.05	4	3.5	**96.5**	0.0	0.0	0.0	5.9	0.2
0.70		0.05	4	0.0	99.4	**0.6**	0.0	0.0	6.0	0.0
1.00		0.50	4	0.0	20.6	**79.4**	0.0	0.0	6.6	0.6
1.10		0.50	4	0.0	14.1	85.9	**0.0**	0.0	6.4	0.5
2.50		1.00	4	0.0	0.0	8.2	**91.8**	0.0	6.2	0.3
10.00		5.00	4	0.0	0.0	0.0	20.6	**79.4**	6.6	0.6
0.03		0.05	10	**100.0**	0.0	0.0	0.0	0.0	1.1	1.1
0.15		0.05	10	0.0	**100.0**	0.0	0.0	0.0	6.0	0.0
0.70		0.05	10	0.0	100.0	**0.0**	0.0	0.0	6.0	0.0
1.00		0.50	10	0.0	0.1	**99.9**	0.0	0.0	6.0	0.0
1.10		0.50	10	0.0	0.0	100.0	**0.0**	0.0	6.0	0.0
2.50		1.00	10	0.0	0.0	0.0	**100.0**	0.0	6.0	0.0
10.00		5.00	10	0.0	0.0	0.0	0.1	**99.9**	6.0	0.0
LC50 for females 10 times greater than LC50 for males										
LC50 (M)	LC50 (F)									
0.03	0.30	0.05	4	**0.1**	99.9	0.0	0.0	0.0	6.0	0.0
0.15	1.50	0.05	4	0.0	**81.9**	18.1	0.0	0.0	6.2	0.0
0.70	7.00	0.05	4	0.0	0.6	**98.1**	1.3	0.0	7.0	0.0
1.00	10.00	0.50	4	0.0	0.0	**94.4**	5.6	0.0	6.1	0.0
1.10	11.00	0.50	4	0.0	0.0	92.3	**7.7**	0.0	6.1	0.0
2.50	25.00	1.00	4	0.0	0.0	0.0	**88.6**	11.4	6.1	0.0
10.00	100.00	5.00	4	0.0	0.0	0.0	0.0	**100.0**	6.0	0.0
0.03	0.30	0.05	10	**0.0**	100.0	0.0	0.0	0.0	6.0	0.0
0.15	1.50	0.05	10	0.0	**98.7**	1.3	0.0	0.0	6.0	0.0
0.70	7.00	0.05	10	0.0	0.0	**100.0**	0.0	0.0	7.0	0.0
1.00	10.00	0.50	10	0.0	0.0	**100.0**	0.0	0.0	6.0	0.0
1.10	11.00	0.50	10	0.0	0.0	100.0	**0.0**	0.0	6.0	0.0
2.50	25.00	1.00	10	0.0	0.0	0.0	**99.9**	0.1	6.0	0.0
10.00	100.00	5.00	10	0.0	0.0	0.0	0.0	**100.0**	6.0	0.0

LC_50_, median lethal concentration.

Performance was assessed both with and without a gender difference in the sensitivity of rats to acute inhalation toxicity. In order to evaluate the classification properties of each procedure in the presence of a gender difference, the LC_50_ values of the less sensitive gender were assumed to be 10 times larger than those in the more sensitive gender.

## Results

### Assessment of gender differences in sensitivity to acute inhalation exposure

Estimated LC_50_ values for males and females were obtained for 56 studies. In the remaining studies, the probit regression models failed to converge. This means that model parameters and, therefore, LC_50_ values could not be estimated. In some cases, failure to converge was due to the small size of the study, for example two concentration levels with five males and five females tested at each level. In other cases, none of the animals tested at or below a given concentration level died, whereas all of the animals tested at or above the next highest concentration level died, leading to a complete separation of the response variable, death. In such cases, a range of concentration levels provide an equally good (perfect) fit to the data with an infinitely steep concentration-response curve. The estimation of the model parameters therefore breaks down and the model fails to converge, making it impossible to estimate the LC_50_.

Statistically significant differences between the log_10_ LC_50_ values for males and females were observed in 16 of the 56 studies (29%) for which the probit regression model converged, each corresponding to a different substance. The results are summarized in Table 2 , which shows the number of animals (male and female) in each of the 16 studies, estimated log_10_ LC_50_ values for males and females with 95% confidence intervals, and the *p* value for the test of a gender effect on the probability of death. The estimated LC_50_ values for males and females differed mainly less than 10-fold. There was a more than 10-fold difference for two substances; ammonia had an estimated LC_50_ for females 12 times that for males and borax (99.51%) had an estimated LC_50_ for males 19 times that for females. Both males and females were found to be more sensitive: in 11 out of the 16 studies where a significant difference was found, females were found to be more sensitive than males to acute inhalation exposure.

**Table 2.** Estimated log10 LC50 values for males and females for 16 substances

Substance	No. of animals		*p* Value	True Male estimated log_10_ LC_50_ (95% CI)^a^		Female estimated log_10_ LC_50_ (95% CI)^a^		
	M	F						
Acetaldehyde	20	20	0.015	1.455	(1.377, 1.532)	1.318	(1.233, 1.404)
Acrylonitrile	40	40	0.007	0.054	(0.023, 0.085)	0.123	(0.098, 0.148)
Ammonia	100	100	<0.001	0.714	(0.147, 1.282)	1.796	(1.199, 2.394)
Arsine	180	180	<0.001	–0.110	(–0.245, 0.025)	–0.594	(–0.723, –0.464)
Bensulide (65.88%)	15	15	0.024	0.480	(0.364, 0.595)	0.280	(0.166, 0.393)
Bioallethrine (93.0%)	15	15	0.030	0.567	(0.395, 0.737)	0.269	(0.096, 0.442)
Borax (99.51%)	15	15	0.022	1.409	(–0.404, 3.222)	0.133	(–0.558, 0.824)
Chlorothalonil (14.7%)/ diuron (19%)	15	15	0.008	–0.066	(–0.336, 0.203)	–0.483	(–0.748, –0.217)
Chlorothalonil (75%)	25	25	0.024	–1.285	(–1.511, –1.060)	–1.678	(–1.904, –1.449)
Copper ammonium carbonate (22.8%)/bardac 22 (4.7%)	25	25	0.044	0.174	(0.027, 0.321)	0.391	(0.250, 0.531)
Copper hydroxide (17.1%)/copper sulfate pentahydrate (26.29%)	15	15	0.035	0.092	(–0.021, 0.205)	0.265	(0.154, 0.375)
Ethylene oxide	15	25	0.003	1.030	(1.003, 1.057)	0.872	(0.853, 0.891)
Idomethane (25%)/chlorpicrin (75%)	40	40	0.037	–0.822	(–0.980, –0.664)	–0.618	(–0.746, –0.491)
Phorate (20%)	25	25	0.024	–1.073	(–1.212, –0.934)	–1.364	(–1.671, –1.067)
Rotenone (45%)	20	25	0.007	–2.013	(–2.131, –1.895)	–2.230	(–2.322, –2.139)
Ziram (50%)/ 2-mercaptobenzothiazole, zinc salt (4%)	15	15	0.013	–0.771	(–1.178, –0.364)	–1.747	(–2.071, –1.423)

LC_50_, median lethal concentration; CI, confidence interval.

^a^ LC_50_ in mg/L

### Comparison of test methods

The results of the statistical evaluations for the three test procedures are summarized in Figures 5–8 and Tables 3–6. The figures show the probability of classification into each toxic class for a range of hypothetical substances in the dusts and mists category with LC_50_ values ranging from 0.01 to 50 mg/L. For each LC_50_ value (plotted across the bottom of the graph), the first vertically sloping line shows the probability (using the scale on the left hand axis) of classification into class 1, the second into class 1 or 2 (so that the difference between this and the one below is the probability of classification into class 2), the third into class 1, 2 or 3 (so that the difference between this and the one below is the probability of classification into class 3) and so on. The vertical dotted lines give the correct classes, and the dashed lines horizontally across the plots show the expected number of animals and deaths (using the scale on the right-hand axis), with the top line indicating the number of animals used. For each LC_50_ value, the height of the shaded areas gives the probability of correct classification, the height of the area below the shaded area is the probability of classification into too toxic a class (impossible for true class 1) and the height of the area above the shaded area is the probability of classification into a class that is not toxic enough (impossible for true class 5). Classification is generally more accurate when the concentration-response curve is steep, and figures corresponding to a concentration-response curve slope of both 4 and 10 are shown. For TG 403 and the ATC method, the starting concentration makes little difference to the classification probabilities, so that only results for a starting concentration of 5 mg/L are shown (Figures 5 and 6). It should be noted, however, that the number of animals required does depend on the starting concentration, since many more animals are needed if testing starts at a concentration far from the true LC_50_ value.

The tables give classification probabilities and expected numbers of animals and deaths for hypothetical substances in the dusts and mists category, with LC_50_ values 0.03, 0.15, 0.7, 1, 1.1, 2.5 and 10 mg/L and concentration-response curve slope values of 4 and 10. The starting concentration in this case was the test concentration assumed to lead to death or evident toxicity in some of the animals. For the FCP, *R* values of 5 and 50 were considered. The probabilities of classification into the correct GHS class based on the true LC_50_ value are shown in bold.

The figures and tables show that, as expected, performance is generally poorer for substances with shallower concentration-response curve slopes, with classification being more variable. Although not shown, similar results were obtained for the case of different toxicity and lethality concentration-response curve slopes, with classification probabilities falling between those for the two slope values.

### Properties of TG 403

Classification probabilities and the expected numbers of animals and deaths required for classification using TG 403 are shown in Figure 5 and Table 3. Considering first the results in the absence of a gender difference, it can be seen that, using TG 403, the probability of classification into the correct GHS toxic class is generally high. For the hypothetical substances considered in Table 3, the probability of correct classification is at least 60% for all substances except those with an LC_50_ value of 1.1 mg/L and a concentration-response curve slope of 4. According to its LC_50_ value, this substance should be placed into class 4, but is very close to the boundary with class 3. This LC_50_ value, together with the shallow concentration-response curve slope, makes classification of this substance particularly difficult, resulting in a probability of correct classification of 38%. When the concentration-response curve slope is equal to 10, with the exception of the substances with an LC_50_ value of 1 mg/L and 1.1 mg/L for which the probabilities of correct classification are 75% and 61%, respectively, the probability of correct classification for the substances considered is at least 99%.

The high probability of correct classification by TG 403 is also shown in Figure 5, which has a large shaded area. Incorrect classification is most likely when substances have an LC_50_ close to the boundary of a toxic class and classification into the adjacent class is possible. Classification of the least toxic substances from a class into the adjacent lower (i.e. less toxic) class is possible but is slightly less likely than classification of the most toxic substances from a class into the adjacent higher (i.e. more toxic) class.

Both Table 3 and Figure 5 show that the number of animals required by TG 403 is large. Since 10 animals are required at each concentration, and testing is required at two concentrations in order to make a classification into classes other than 1 and 5, at least 20 animals are required for many substances even if an appropriate starting concentration is selected. The maximum number of animals required is 40 and the minimum is 10.

In order to consider how the classification of dusts and mists using TG 403 is affected if one gender is more sensitive to acute inhalation toxicity than the other, it is assumed that the LC_50_ for females is 10 times greater than the LC_50_ for males. However, since males and females are treated identically in the procedure, the results would be identical if the LC_50_ for males was 10 times that for females. For substances with LC_50_ values near the middle of their class, the probability of correct classification is largely unchanged and remains high. The gender difference has a greater impact on substances near the class boundaries. Since the probability of death is now lower in the less sensitive gender, there is a greater chance of classification into a less toxic class. As such, the most toxic substances in a class are more likely to be classified correctly while the least toxic substances in a class are more likely to be classified incorrectly into a less toxic class, as shown in Figure 5. This can be seen in Table 3 for the substances with an LC_50_ of 1 mg/L and 1.1 mg/L and dose-response curve slope of 4. The probability of correct classification is increased to 62% for the latter and decreased to 49% for the former. Incorrect classification, if it occurs, is therefore more likely to be into a less stringent class than the true class.

### Properties of the ATC method

Classification probabilities and the expected numbers of animals and deaths required for classification using the ATC method are shown in Figure 6 and Table 4. When there is no difference in the sensitivity of males and females to acute inhalation exposure, the probability of classification into the correct GHS class is high for most substances. The exception to this is the less toxic substances in each class, which are more likely to be assigned to a less stringent class than that suggested by the LC_50_ value, particularly when the concentration-response curve is shallow. This is illustrated by the results in Table 4, where the probability of correct classification for a substance with an LC_50_ of 1.1 mg/L and a slope of 4 is 76%, considerably higher than for TG 403, but the probability of correct classification for a substance with an LC_50_ of 1 mg/L and a slope of 4 is only 34%. Since misclassification, if it occurs, is likely to be considered more serious from a public health perspective if substances are classified into a less toxic class than if substances are classified into a more toxic class, the classification properties of TG 403 would probably be considered more desirable than those of the ATC method. However, this does not take the number of animals required into account. Since the ATC method requires 6 animals per concentration, the minimum number of animals required is 6 and the maximum is 24. With testing typically occurring at two or three concentration levels, the number of animals for most substances, except those in classes 1 and 5, is between 12 and 18.

Now considering the effect of a gender difference in the sensitivity of rats to acute inhalation toxicity, as for TG 403, since males and females are treated identically, the results would be the same whether males or females are more sensitive. The presence of a gender difference means that the chance of seeing death in three of the six animals at the starting concentration is reduced. This leads to an increased likelihood of further testing at higher concentrations and the procedure becomes even less stringent. Substances belonging to class 3 are most affected by the reduced stringency of the method. In order for a substance to be assigned to class 3, the death of at least four animals must be observed at 1 mg/L. Since the chance of seeing death in an animal of the less sensitive gender at 1 mg/L is unlikely without seeing death of all three animals of the more sensitive gender at 0.5 mg/L, observing four deaths is highly unlikely. Classification into class 3 therefore occurs with very small probability, particularly when the dose-response curve is steep, with almost all substances in class 3 assigned to class 4.

### Properties of the FCP

Classification probabilities and the expected numbers of animals and deaths required for classification using the FCP are shown in Figures 7 and 8 (for sighting study starting concentrations of 5 mg/L and 0.05 mg/L, respectively) and Tables 5 and 6 (for *R*, the ratio of the LC_50_ to the TC_50_, equal to 5 and 50, respectively). The properties of the FCP when there is no difference in the sensitivity of rats to acute inhalation toxicity were explored in detail in Stallard et al.^14^ This section will firstly draw comparisons with the other test methods and secondly assess the performance of the procedure when males are more sensitive than females to acute inhalation toxicity.

When the FCP sighting study starts at 5 mg/L, the procedure depends only on death, with an identical outcome to every test regardless of whether evident toxicity is observed. In contrast, when the sighting study starts at a lower concentration, the observation of evident toxicity can affect the classification, so that in the evaluation, it is necessary also to consider the value of *R*.

Considering first the properties of the FCP when the sighting study starts at 5 mg/L (Figure 7), the probability of correct classification is high other than for the most toxic substances in each class. For these substances, there is a high probability of classification into the adjacent more stringent class, this probability being higher than for either TG 403 or the ATC method. The probability of classification into the adjacent less stringent class for the least toxic substances in each class is, conversely, lower than for either TG 403 or the ATC method, indicating that when misclassification occurs it is more likely to be into a more toxic rather than a less toxic class, so that the procedure is more stringent. The number of animals required is lower than for the ATC and considerably lower than for TG 403. Typically, no more than 10 animals are needed, and the sighting study is effective at limiting the number of animals required even if an inappropriate starting concentration is selected. The minimum number of animals needed to classify most substances is 6 (1 in the sighting study and 5 in the main study), and the maximum is 21 (1 in the sighting study and 20 in the main study), though the use of a separate sighting study makes the use of such a high number of animals extremely unlikely.

When the sighting study starts at a concentration below 5 mg/L, the classification can depend on observation of evident toxicity. If *R* is larger than the ratio of the test concentrations, toxicity is likely to be observed at more than one fixed concentration below the concentration at which death is expected to occur, so that the procedure will lead to an even more stringent classification. This can be seen in Figure 8 and in Table 6. The effect is particularly marked for substances in class 4 with an LC_50_ of 1.1 mg/L due to the closeness of the testing concentrations, 1 mg/L and 0.5 mg/L, below this class. The effect becomes more pronounced as the value of *R* increases.

Unless there is prior evidence of a gender difference, the FCP tests females only. Therefore, if females are more sensitive than males, the results considered above for the situation when there is no gender difference would still apply. If females are less sensitive than males to acute inhalation toxicity, the procedure becomes much less stringent. When the procedure starts at 0.05 mg/L (Figure 8), the test performance is to some extent balanced by the stringency of the test discussed above, particularly for the larger value of *R*.

## Discussion

As part of the process for achieving acceptance of new alternative test methods by the OECD and regulatory bodies around the world, it is generally considered important to demonstrate that the new method will provide at least an equivalent level of protection as the method(s) currently employed for the particular purpose. The current methods, by default, are generally considered to be the ‘gold standard.’ For this reason, a comparison of alternative methods with the currently employed methods is particularly important. In this paper, we have reported a statistical evaluation and characterisation of the performance of TG 403, together with a comparison of this method with more recently developed alternative tests (ATC and FCP), to add to the evaluations of the latter that have been previously reported.^10,14,21^

In addition, previous evaluations of acute inhalation toxicity test methods have not taken into account the potential for differences in the susceptibility of males and females to acute inhalation toxicity. This is important to address given that one of the key differences between the three methods is that, in the absence of prior information indicating the presence of gender differences, TG 403 and the ATC method will be conducted in both males and females, whereas the draft FCP proposes to use only females.

Little useful information has previously been reported on the relative sensitivity of male and female rats in acute inhalation studies.^20^ To address this, we reviewed historical acute inhalation toxicity data included in the 2008 OECD Performance Assessment Report^11^ to assess the potential for gender differences in sensitivity. We found that differences in susceptibility between the genders can indeed arise in some acute inhalation studies, and that males or females may be the more sensitive gender.

In light of this finding, we carried out an additional statistical evaluation of the performance of TG 403, the ATC method and the FCP in the presence of gender differences in the sensitivity of rats to inhalation toxicity, assuming a 10-fold difference in LC_50_ between genders.

TG 403 performs well for the most toxic substances when the concentration response curve is steep, although performance declines slightly as the curve becomes shallower. Misclassification, when it occurs, is more likely to place a substance into a class that is too stringent rather than a class that is not stringent enough. Classification into a less stringent class is also possible, although slightly less likely than over-classification. For the majority of substances, classification using TG 403 is unaffected by gender differences, although there is an increased chance of classifying the least toxic substances from each class into a class that is not stringent enough, particularly when the concentration-response curve is shallow.

The ATC method performs well for the most toxic substances, though misclassification into a less toxic class occurs with higher probability than for TG 403, i.e. the method is less stringent. As with TG 403, the performance of the ATC method declines as the concentration-response curve becomes shallower, with a notable increase in the probability of classification into less stringent classes. Apart from the way in which observation of a gender difference affects subsequent testing, the ATC method is very similar to TG 403, only using fewer animals at each concentration. The relative performance of the methods can thus be seen as an immediate consequence of a change in the number of animals tested. In the presence of gender differences, there is a greater tendency to assign substances incorrectly to a less toxic class. This is particularly true for substances in class 3, almost all of which are classified into the less stringent class 4.

The FCP also performs well when the concentration-response curve is steep, with the exception of class 4 substances that have LC_50_ values on the boundary between classes 3 and 4, where it is likely that those substances will be classified into the more stringent class 3. As with the other methods, the performance generally declines as the concentration-response curve shallows, but the FCP continues to perform well for the most toxic substances. For less toxic substances, as the concentration-response curve becomes shallower, the FCP tends to be over-stringent in its classifications, in contrast to the TG 403 and ATC methods where there is more potential for under-classification. If the sighting study starts at a concentration other than 5 mg/L, the classification depends on evident toxicity in addition to mortality. This means that the classification can be too stringent if evident toxicity is observed at low concentrations.

As the FCP proposes the testing of females only, if males are more sensitive than females the procedure will be much less stringent, particularly when there is a low ratio between the LC_50_ and the TC_50_ (i.e. a low value of *R*), as this results in a lower range of concentrations where non-lethal toxicity rather than mortality will be seen. Indeed, when *R* is equal to 5 and the concentration-response slope is at its steepest, if the LC_50_ for males is one-tenth that for females, the FCP performs poorly for all but the least toxic substances (i.e. those in class 5), with most misclassifications being made into a less stringent class than the true class. In contrast, when *R* is equal to 50, the range of concentrations over which non-lethal toxicity rather than death will be observed is increased. This improves the chances of correct classification and, when the concentration-response curve is steep, the majority of substances are classified into the correct class. As the curve becomes shallower, the disparity in the performance of the FCP for the two *R* values reduces.

Based on these analyses, it is clear that even the traditional LC_50_ test for assessing acute inhalation toxicity does not perform perfectly for all substances. These limitations, together with the inevitable compromise between maximising the probability of correct classification and minimising the number of animals required, need to be taken into account when evaluating alternative methods for this purpose. Clearly, no method is perfect and misclassification is a general problem not specific to any particular test method, particularly for substances with shallow concentration-response curves. There is growing recognition of this and despite the acknowledged limitations of the ATC method,^11^ this has recently been accepted as a validated OECD test method.

Since TG 403 bases classifications on the more stringent result from the male and female testing, in the presence of a gender difference, classifications are based solely on the outcomes for the more sensitive gender. This highlights two points, firstly, it means that the less sensitive gender (females in the evaluation discussed here) is redundant in the classification process and is therefore exposed unnecessarily. Secondly, as the results in Table 3 show, when there is a difference in the sensitivity of males and females to acute inhalation toxicity, TG 403 is less stringent than when both genders have the same LC_50_.

In the absence of gender differences, the FCP tends to be more stringent than the other two methods, with less chance of misclassification into less stringent classes. Although this can be viewed as a disadvantage due to increased economic costs for the chemical and transport industry through the need for stricter controls over the handling of the substance, from a public health protection perspective, over-classification is considered preferable to under-classification. Furthermore, acute toxicity data are not only used for classification and labelling purposes, but can also play a role in risk assessment and communication. A simple estimation of LC_50_ is of little value for assessing potential risk in humans. It has been argued that information on clinical signs of toxicity observed at doses lower than those causing lethality, including the time to onset, duration and rate of recovery, as can be obtained using the FCP, would be of greater value.^15,17^

Given that the draft FCP proposes the use of a single gender only, it is unsurprising that our evaluation has shown impairment in the performance of the FCP in the presence of gender differences in susceptibility to acute inhalation exposure. In light of this, we have recently evaluated the performance of a revised FCP protocol that involves the testing of both genders during the sighting study phase for substances where prior information on gender differences is unavailable.^22^

Our analyses have also demonstrated substantial differences in the number of animals used for each method. The ATC method provides an advantage over the LC_50_ method by using fewer animals (6–24 versus 10–40), while the FCP uses even fewer (2–11). The FCP also provides further benefits in terms of animal welfare by not requiring lethality as an endpoint, and the present work, together with additional activities coordinated by the NC3Rs, will be used to build a robust evidence-based case to support the international adoption of this test.

## Appendix Details of the statistical modelling method

Suppose that a given substance has a probit concentration-response curve with slope β and LC_50_ values *l*_0_ for males and *l*_1_ for females, where each value is assumed known. For a single male or female animal tested at concentration *x*, the probability of death is given by:

(1) $$pr\;(\text{death})\;=\;\Phi \;({\text{β(log}}_{\text{10}}x\;-\;\log_{10}\;l_{i}))$$

where *i* = 0 or 1 for a male or female, respectively. The model assumes that the slope, β, is identical for males and females, which amounts to assuming that there is no interaction between exposure concentration and gender.

If there is no gender difference in the sensitivity of rats to acute inhalation toxicity, *l*_0_ and *l*_1_ are identical, say *l*, and the probability of death is the same for males and females:

(2) $$pr\;(\text{death})\;=\;\Phi \;({\text{β(log}}_{\text{10}}x\;-\;\log_{10}\;l)).$$

For the FCP, it is also necessary to calculate the probability of non-lethal evident toxicity in an exposed animal. Let *R* denote the ratio of the LC_50_ to the TC_50_, where the TC_50_ is the concentration expected to cause death or evident toxicity in 50% of the animals. Then, the TC_50_ is equal to *l_i_, i* = 0 or 1, for males and females, respectively. If the concentration-response curves for toxicity and lethality are assumed to have the same slope, for a single male or female tested at concentration *x*, the probability of death or evident toxicity is

(3) $$pr(\text{death or evident toxicity})\;=\;\Phi \;(\text{β}\;{\text{(log}}_{\text{10}}x\;-\;\log_{10}\;(l_{i}/R)),\;i\;=\;0\text{ or }1.$$

The probability of non-fatal evident toxicity for a single male or female animal is then obtained by subtracting (1) from (3), and the probability of neither death nor evident toxicity is calculated as 1 -. A model with different slopes in (1) and (3) can also be assumed, although in this case it is necessary to take the probability of death or evident toxicity to be the maximum of the right-hand sides of (1) and (3) to ensure that the curves do not cross.

Using (1), and (3) in the case of the FCP, the probabilities of classification into each of the GHS classes for a substance with known toxicity properties, that is known LC_50_ and concentration-response curve slope, can be obtained by considering all possible test sequences. The probability of a given substance being assigned to each GHS class can then be calculated. Given the probability of each classification, the probability of the correct classification, given the LC_50_ value, can be deduced. In the case of a gender difference, the correct classification is taken to be that corresponding to the LC_50_ value for the more sensitive gender.
